# Unique E2-binding specificity of artificial RING fingers in cancer cells

**DOI:** 10.1038/s41598-024-52793-y

**Published:** 2024-01-31

**Authors:** Kazuhide Miyamoto, Takashi Tadokoro, Atsushi Matsumoto

**Affiliations:** Faculty of Pharmaceutical Sciences, Sanyo-Onoda City University, 1-1-1 Daigaku-dori, Sanyo-Onoda, Yamaguchi 756-0884 Japan

**Keywords:** Enzymes, Ubiquitylation

## Abstract

Ubiquitin (Ub)-conjugating enzymes (E2s) are involved in various pathways for Ub transfer and deubiquitinating activities. These enzymes are associated with cancers such as breast cancer which is the second deadliest type of malignancy among women. Here, we revealed the unique E2-binding property and the auto-ubiquitination of artificial RING fingers (ARFs). Circular dichroism spectra showed the characteristic structures of ARFs. The proline, lysine, leucine, threonine and cysteine (PKLTC) sequence of ARF was important for E2-recognition and its mutations induced obvious changes in the E2-binding specificity and the auto-ubiquitination activity of ARF. The ARF mutants were applicable to detection of most of E2 activities. Furthermore, adding the ARF mutant C35A to cancer cells promoted its auto-ubiquitination, leading to the preferential detection of E2 UbcH5b activity. The present work opens up a new avenue for investigating intracellular E2 activities for the fatal diseases.

## Introduction

Protein ubiquitination is a eukaryotic cellular process and is one of the most important post-translational modifications^1,2^. Ubiquitination is a complicated enzymatic cascade system, consisting of ubiquitin (Ub)-activating (E1), -conjugating (E2), and -ligating (E3) enzymes. The amounts of Ub transferred from E2s onto a substrate are referred to as E2 activities during ubiquitination^3^. E3s, in general, grab their substrates and partner E2s, and then, Ub is transferred from an E2 to various types of substrates^4^. E2-Ub conjugates have opened and closed conformations to facilitate Ub transfer from E2 to substrates^5^. Not only do E2s transport Ub, they also play essential roles in ubiquitination reactions. For example, E2s are involved in determining where and how Ub attaches to substrates^6^. Increasing evidence suggests that E2s accumulate in various types of cancer^7–10^ as well as specific neurological disorders^11^, and are potential biomarkers for diagnostic and prognostic purposes^12^. Approximately 40 E2s transfer Ub for protein functions and stability in humans^6^. An E3 interacts with some E2s and develops the ubiquitination of some substrates^13^, and thus, estimating E2 activities as amounts of Ub attached to all substrates is challenging. Artificial RING fingers (ARFs) bind to E2s and are ubiquitinated by E2s without the substrates^14^. ARFs are useful to detect E2 activities, and interact with some types of E2s, e.g., UbcH5a, b, and c which share their high sequence similarities. However, specific detection of various E2 activities remains a major challenge. To expand the use of ARFs in cancer cells, we report a unique E2-binding property of ARFs. Breast cancer is the second most common malignancy among women^15^. We derived ARFs from E3s associated with breast cancer metastasis.

## Materials and methods

### Peptide synthesis

**Figure 1:**
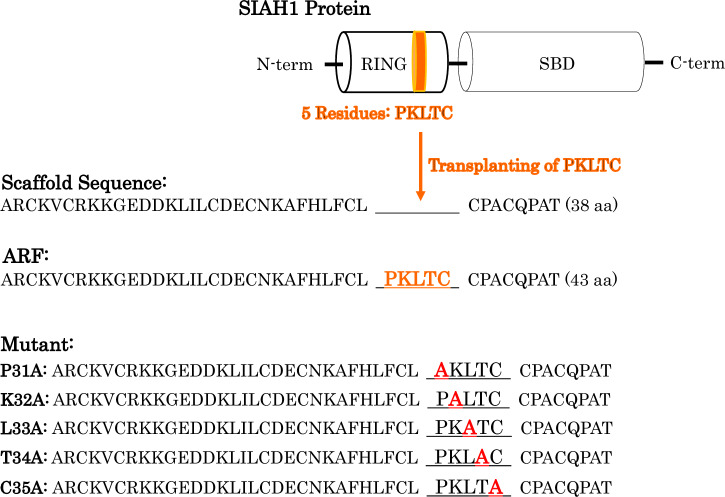
Molecular design of artificial RING finger (ARF). We engineered ARF as a chimeric finger using the PKLTC sequence of the SIAH1 RING finger transplanted on the WSTF PHD finger as a scaffold. Five mutants of ARF were designed by replacing the PKLTC sequence with Ala (red).

We created ARFs by transplanting proline, lysine, leucine, threonine and cysteine (PKLTC) of human seven in absentia homolog E3 Ub protein ligase 1 (SIAH1) RING into a scaffold amino acid sequence from the Williams-Beuren syndrome transcription factor (WSTF) plant homeodomain (PHD) zinc finger (Fig. 1)^16^. Replacement of PKLTC with Ala resulted in five ARF mutants that were synthesized along with wild-type ARF using the standard Fmoc solid-phase method. Chemicals for peptide assembly and amide resin were purchased from HiPep Laboratories (Kyoto, Japan). After cleavage with trifluoroacetic acid, synthesized peptides were purified by reversed-phase high performance liquid chromatography (HPLC) using a Kinetex 5-μm EVO C18 column (Phenomenex, Torrance, CA, USA). The peptides were > 98% pure, and their molecular masses were confirmed by matrix-assisted laser desorption ionization-time of flight mass spectroscopy (MALDI-TOF MS) using an AXIMA-TOF2 mass spectrometer (Shimadzu Corp., Kyoto, Japan). The peptide was dissolved and denatured in 1 mL of 8 M guanidine-HCl, then dialyzed at 4 °C overnight against degassed 20 mM Tris–HCl (pH 6.8), 50 mM NaCl, 1 mM dithiothreitol (DTT), 50 μM ZnCl_2_, and 10% glycerol (Solution A) using Slide-A-Lyzer dialysis cassettes (Thermo Fisher Scientific Inc., Waltham, MA, USA)^16^.

### Auto-ubiquitination of ARF in vitro

Ubiquitination proceeded in 20 mM Tris–HCl (pH 6.8) containing 5 mM Mg-ATP, 1 mM DTT, 20 U/mL inorganic pyrophosphatase (Sigma-Aldrich Corp., St. Louis, MO, USA), and 50 μM ZnCl_2_. The reaction mix contained 2.5 μM randomly biotinylated Ub, as well as His-tagged human recombinant E1 (0.1 μM) and 1.0 μM each of the E2s UbcH1, 2, 3, 5a, 5b, 5c, 6, 7, 8, 10, and 13/Mms2 (Enzo Life Sciences, Farmingdale, NY, USA). Thereafter, ARF or mutants (10 μM) were added to the solution and gently mixed. The reactions were incubated at 37 °C for 60 min, then quenched with non-reducing sodium dodecyl sulfate (SDS) buffer. The samples in MES buffer were separated using NuPAGE™ (12% Bis–Tris gel; Thermo Fisher Scientific Inc., Waltham, MA, USA), then transferred to polyvinylidene difluoride (PVDF) membranes. Biotinylated ubiquitinated products were detected using an avidin–biotin based peroxidase system (Vectastain ABC Elite Kits; Vector Laboratories, Burlingame, CA, USA). Signals enhanced using ECL Reagents (Amersham, Little Chalfont, UK) were detected using a WSE-6100 LuminoGraph I luminescent image analyzer (ATTO, Tokyo, Japan).

### Cell culture

We seeded MCF7 cells, kindly provided by Dr. Yoshihiro Kuroda (Himeji Dokkyo University, Himeji, Japan), into 35 mm dishes containing Dulbecco’s modified Eagle’s medium (DMEM), supplemented with 10% fetal bovine serum (FBS), 100 units/mL of penicillin, and 100 mg/mL of streptomycin (DMEM/10% FBS) and incubated them at 37 °C under a 5% CO_2_ atmosphere until they reached 80% confluence. The medium was removed, then the cells were incubated for 9 h in DMEM without FBS.

### Detection of ARF reactivity in cells

We detected ARF reactivity in cancer cells using the ARF mutant C35A (replacement of C35 with Ala). C35A (1.0 mg) was randomly biotinylated with 0.17 mg succinimidyl biotin (ProteoChem, Hurricane, UT, USA) in phosphate buffer (pH 8.0) for 1 h at room temperature. Biotinylated C35A was dialyzed against 8 M guanidine-HCl, followed by Solution A. The obtained C35A solution was added to MCF7 cells in DMEM along with 40 μM of the cell-penetrating peptide L17E (a kind gift from Dr. Shiroh Futaki (Kyoto University, Kyoto, Japan))^17^, and incubated at 37 °C for 1 h. The cells were washed with cold PBS, scraped into 15 mL tubes, and centrifuged at 2000 rpm for 5 min. Pelleted cells were suspended in an equal volume of 20 mM Tris–HCl (pH 8.5) containing 1% Triton X-100, 2 mM MgCl_2_, and 12.5 U/mL Benzonase (Sigma-Aldrich), then placed for 5 min on ice. The cells were lysed in 1:10 volumes of 10% SDS and heated at 70 °C for 5 min. Protein concentrations were determined using Quick Start™ Bradford assays (Bio-Rad Laboratories Inc., Hercules, CA, USA). Proteins (20 μg) from cell extracts were separated using NuPAGE as described above with MES buffer, then transferred onto a PVDF membrane. Biotinylated C35A was detected on the membranes by incubation for 1 h with horseradish peroxidase streptavidin of Vectastain ABC Elite Kits. Enhanced signals were captured using a WSE-6100 LuminoGraph I. Actin (control) was assessed using anti-actin (sc-1616) and anti-goat-IgG (sc-2020; both from Santa Cruz Biotechnology Inc., Dallas, TX, USA). Silencer™ Select Negative Control #1 small interfering RNA (siRNA, 4390843), Validated siRNA for UbcH5b (s14576) and Lipofectamine™ RNAiMAX transfection reagent were used for knockdown experiments (Thermo Fisher Scientific Inc., Waltham, MA, USA). UbcH5b siRNA knockdown in MCF7 cells was performed according to the manufacture’s instruction. Randomly biotinylated C35A (25 μM) was incubated in UbcH5b-knockdown cells. The obtained cell lysates were separated by Tris–Glycine SDS-PAGE. The reactivity of biotinylated C35A was detected with horseradish peroxidase streptavidin (SA00001-0, Proteintech Group, Inc., Rosemont, IL, USA).

### Structure modeling

The structure of the ARF model was calculated using Iterative Threading Assembly Refinement (I-TASSER), which is ranked as the top server in Critical Assessment of Structure Prediction (CASP)^18^. Templates for structure prediction and structure-based function annotations were automatically selected from Protein Data Bank using Local Meta-Threading Server (LOMETS) v. 3.0^19^. Continuous fragments of templates were reassembled into full-length structures by a Monte Carlo-based simulation algorithm using the replica-exchange method.

### Circular dichroism (CD) spectroscopy

We calibrated a J-805 spectropolarimeter (JASCO Corporation, Hachioji, Japan) using d-camphor-10-sulfonate. Thereafter, CD spectral data from samples (25 μM) in Solution A were acquired using quartz cells with a 1-mm path length. The spectra were collected under the following conditions: room temperature; wavelength, 200‒250 nm; bandwidth, 1 nm; data pitch, 1 nm; scan speed, 50 nm/min^20,21^. Each CD spectrum was measured as the average of four scans, and CD data were obtained by transforming CD signals into mean residue molar ellipticity.

## Results and discussion

### Auto*-*ubiquitination assays of ARFs

**Figure 2:**
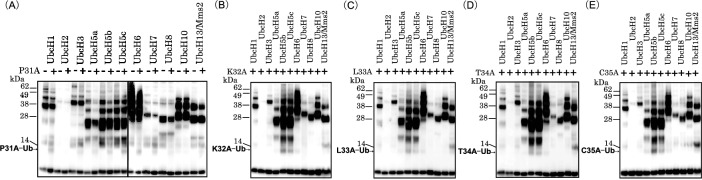
Substrate-independent assays of artificial RING finger (ARF) ubiquitination in vitro. ARF mutants ((**A**) P31A, (**B**) K32A, (**C**) L33A, (**D**) T34A, and (**E**) C35A) were incubated with randomly biotinylated ubiquitin (Ub), recombinant E1, and 11 E2s. ARF mutants promoted the addition of Ub chains to itself. The reactions were resolved using NuPAGE. Ub chains on PVDF membranes were visualized using Vectastain ABC Elite Kits. Emitted signals were detected using WSE-6100 LuminoGraph I.

We transplanted the RING domain of SIAH1 that is associated with metastatic breast cancer to create ARFs^22^. The wild-type ARF interacts with E2s (UbcH5a, 5b, 5c, 6, and 8) and is mono-ubiquitinated by E2s^14^; thus, to specifically detect an E2 of interest using the wild-type ARF is impossible. It remains to be seen whether and how E2 specificity of ARF is regulated during ubiquitination. If the amino-acid sequence of PKLTC in ARF indeed contributes to its formation of the E2-binding site, the amino acid replacement of PKLTC must affect the E2 specificity of ARF. To address this issue, we assessed whether the five mutants of ARF (P31A, K32A, L33A, T34A, and C35A, shown in Fig. 1) have ubiquitination activity and E2 specificity using substrate-independent ubiquitination assays in vitro. Ub thioester-linked E2 conjugates (E2-Ub conjugates) form during the ubiquitination of Ub, E1, and E2^23^. Thereafter, adding ARFs to the ubiquitination reaction led to the formation of ARF-Ub conjugates where Ub was transferred from E2-Ub conjugates (Fig. 2 and Supplementary Fig. S1). Such reactions displayed the auto-ubiquitination of ARFs without substrates^16^. Surprisingly, the P31A mutant cooperated with UbcH1, 3, 5a, 5b, 5c, 6, 7, 8, and 13/Mms2, and then promoted the development of P31A-Ub conjugates. We found that P31A interacted with most of E2s under our conditions, which was a unique property not previously reported among E3s. The K32A, L33A, and T34A mutants were mono-ubiquitinated in the presence of UbcH5b and 5c, which had similar ubiquitination activities and E2 specificities. However, a strong band corresponded to C35A-Ub conjugates when C35A was incubated only with UbcH5b, whereas the bands for UbcH5a, UbcH5c, and UbcH13 were very weak. C35A enabled the preferential detection of UbcH5b activity. The di-ubiquitinated products of C35A were also observed in Supplementary Fig. S2. All ARF mutants were clearly mono- or di-, and not poly-ubiquitinated. Taken together, these data revealed that point mutations of the PKLTC sequence in ARF resulted in obvious changes in its E2 specificity. Therefore, it was found that the PKLTC sequence is one of control factors for regulating E2 specificity. The structural features of ARF at atomic level will be examined later via structural modeling methods.

### Detection of ARF reactivity in cancer cells

**Figure 3:**
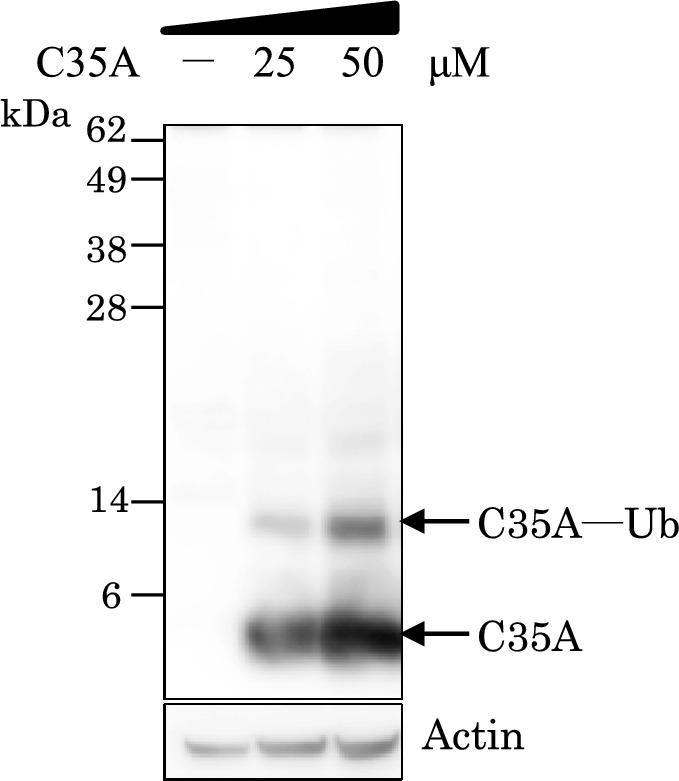
Reactivity of artificial RING finger (ARF) in cancer cells. Biotinylated ARF mutant C35A (0, 25, and 50 μM) was incubated with MCF7 cancer cells and endosomolytic peptide L17E, then cell lysates were resolved by NuPAGE. Ubiquitinated C35A was assessed by western blotting and emitted signals detected using WSE-6100 LuminoGraph I were compared with those of actin (control).

**Figure 4:**
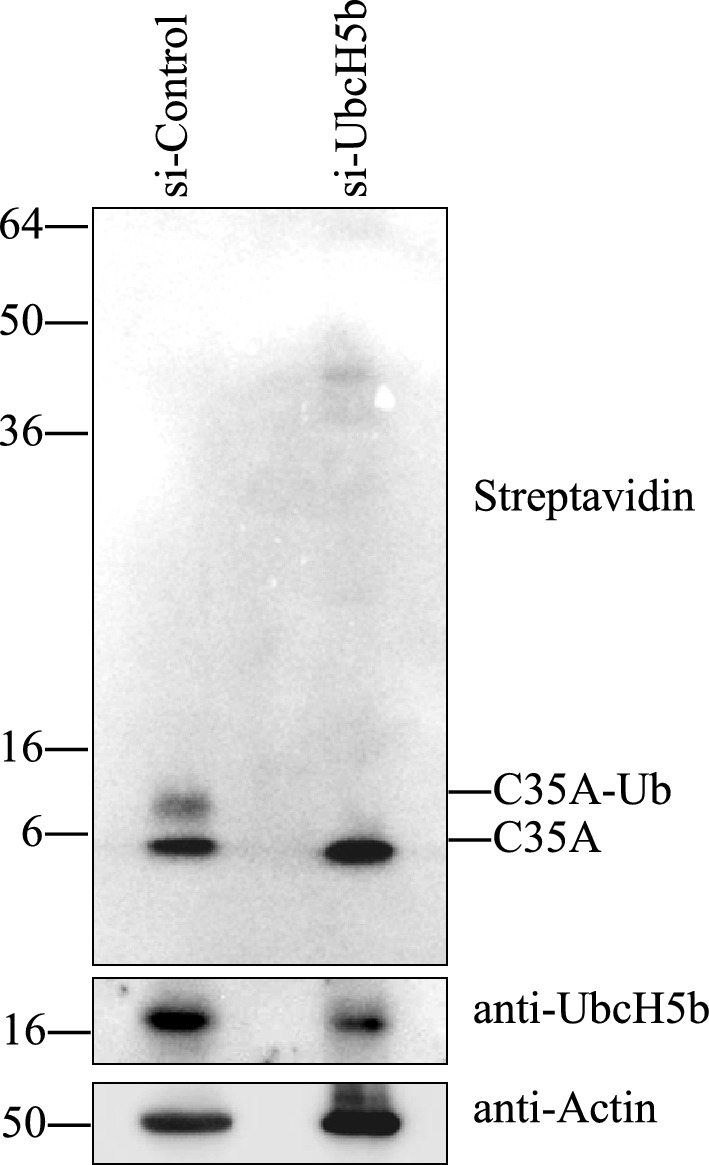
UbcH5b knockdown inhibits the ubiquitination of C35A in cancer cells. Biotinylated ARF mutant C35A (25 μM) was incubated with the peptide L17E in MCF7 cancer cells after the siRNA of UbcH5b was transfected. The silencing of UbcH5b decreased the level of the ubiquitination of C35A. Actin and UbcH5b were assessed as controls using goat anti-actin (sc-1616) and rabbit anti-UbcH5b (bs-8348, Bioss antibody), respectively.

We assessed the uptake and reactivity of the C35A mutant in MCF7 cells, since it had the highest E2-binding specificity among the five mutated ARF constructs using ubiquitination assays in vitro. The lipid-sensitive endosomolytic peptide L17E was used to penetrate cells. This peptide enhances the uptake of biomolecules such as cytosolic proteins and antibodies delivered via the induction of micropinocytosis^17^. After incubating cells with L17E and the biotinylated ARF C35A, reactivity was detected by western blotting (Fig. 3 and Supplementary Fig. S3). The products of mono-ubiquitinated ARF (C35A-Ub) were the most prevalent after 1 h of incubation with C35A. Moreover, reactivity was concentration-dependently augmented in cells incubated with C35A (0‒50 μM). The L17E peptide did not abolish intracellular ARF reactivity. These data displayed C35A uptake into MCF7 cells and intracellular ubiquitination activities. The ubiquitination of C35A meant that Ub was principally transferred from UbcH5b, that is, preferential UbcH5b activity. To further confirm whether the ubiquitination of C35A was developed by cooperating with UbcH5b in cells, UbcH5b expression was silenced by UbcH5b siRNA. Knockdown of UbcH5b reduced the level of the C35A ubiquitination relative to the control siRNA (si-Control) as shown in Fig. 4 (Supplementary Fig. S4). Although the poly-Ub chains, in general, are the target signal for the proteasomal degradation, the mono-ubiquitination of SIAH1 promotes the aggregation of alpha-synuclein^24^. UbcH5b activities were predominantly detected by C35A in cancer cells for at least 1 h.

Both UbcH5b and 5c are associated with the initiation and progression of human cancers and immune disorders^12,25,26^ and UbcH5c associated with NF-κB activity is overexpressed in some types of human cancer^26^. Sequence alignment of the E2s UbcH5a, 5b, and 5c showed the importance of residues 7, 11, and 138 (Supplementary Fig. S5). Only these residues were not conserved between UbcH5b and 5c. Some or all of these positions contributed to the formation of the C35A-UbcH5b/c complex. Because the UbcH5 family members were all similar, evaluations of E2s might have been limited due to the inability of small-molecule inhibitors to detect the inhibitory effects of either UbcH5b or 5c^26^. This study showed the first detection of UbcH5b activities in cancer cells. However, given the importance of UbcH6 in the development of the fatal diseases such as autosomal-dominant neurodegenerative^27^, the detection of the only UbcH6 activity is desirable. The P31A mutant enabled simultaneous E2-detection of 9 E2s under the present conditions. We will collect various ARFs to achieve specific E2 detections in the future, e.g., E2 screening.

### Structure of ARFs

**Figure 5:**
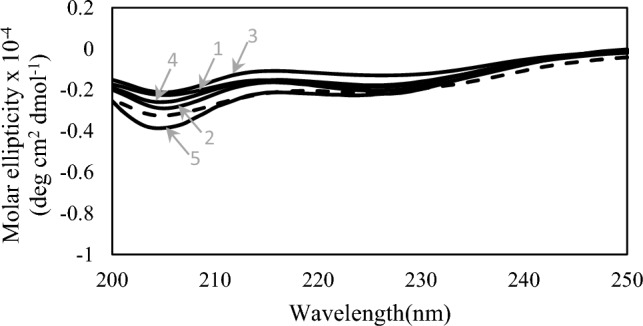
CD spectra of artificial RING finger (ARF) and mutants. Spectra of wild-type and mutant ARFs (1) P31A, (2) K32A, (3) L33A, (4) T34A, and (5) C35A in 20 mM Tris–HCl (pH 6.8) containing 50 mM NaCl, 1 mM DTT, 50 μM ZnCl_2_, and 10% glycerol. Dotted line, wild-type ARF.

The three-dimensional structure of ARF from the present amino-acid sequences remains unknown. The structural homology of the ARF model was calculated using I-TASSER^18^ (Supplementary Fig. S6 and Data S1). The generated structure had high confidence (C-score, 0.77) of threading template alignments and structure assembly simulations, and the accuracy of structure modeling had a TM score of 0.78. The WSTF-PHD (PDB code: 1F62), scyllo-inositol dehydrogenase (PDB code: 5YAB), and UHRF1 (PDB code: 3ASK) templates for modeling calculations were automatically selected by LOMETS^19^. Identity between the ARF sequence and those of the WSTF-PHD, scyllo-inositol dehydrogenase, and UHRF1 templates was 88%, 44%, and 42%, respectively. The ARF compact structure had a ββ arrangement. RING-type E3 ligases possess a hydrophobic groove for E2-binding^28,29^. Like the RING structures, the PKLTC sequence formed the hydrophobic surface on ARF for possible E2-binding. The CD spectra were acquired to estimate the secondary structures of ARF and its five mutants (P31A, K32A, L33A, T34A, and C35A) in Solution A (Fig. 5). These spectra were similar insofar as they all had double-negative minima at ~ 205 and 230 nm (π–π* and n–π* transitions, respectively). Any Ala mutations in the PKLTC region did not cause the ARF structure into collapse, supporting that the mutants have the ubiquitination activities, like the wild-type. Intriguingly, the replacement of Pro31 with Ala did not induce the obvious structural changes of ARF, although its mutant had the unique ubiquitination activity as described above. Accordingly, Pro31 contributed to E2 recognition, but hardly the stability of the whole structure. Thus, the position of Pro31 is a significant key for specific recognition. In contrast, the molecular ellipticity of C35A increased slightly compared with other mutants. The structural different of C35A probably warrants its preferential UbcH5b-binding. Although the present findings provided novel insights into the creation of ARFs, further structural investigation of the ARF-E2 complex will be needed to clarify its binding mechanism.

In conclusion, the unique E2-binding property and the ubiquitination activity of ARFs were demonstrated with auto-ubiquitination assays. The PKLTC sequence is important for E2-recognition of ARF and its point mutations cause obvious changes in E2-binding specificity of ARF. The ARF mutants are applicable to detection of most of E2 activities used here. Furthermore, adding the ARF mutant C35A to live cells promotes its auto-ubiquitination, leading to preferential detection of UbcH5b activity. This work opens up a new avenue for investigating intracellular E2 activities for the fatal diseases.

### Supplementary Information

Supplementary Figure S1.

Supplementary Figure S2.

Supplementary Figure S3.

Supplementary Figure S4.

Supplementary Figure S5.

Supplementary Figure S6.

Supplementary Information.

## Data Availability

All data generated or analyzed during this study are included in this published article and its supplementary information files.
