# Supplementary material for: Unique E2-binding specificity of artificial RING fingers in cancer cells
Source: Sci Rep. 2024 Jan 31;14:2545. doi: 10.1038/s41598-024-52793-y (PMC10828389; doi:10.1038/s41598-024-52793-y)

Supplementary Fig. S1

A

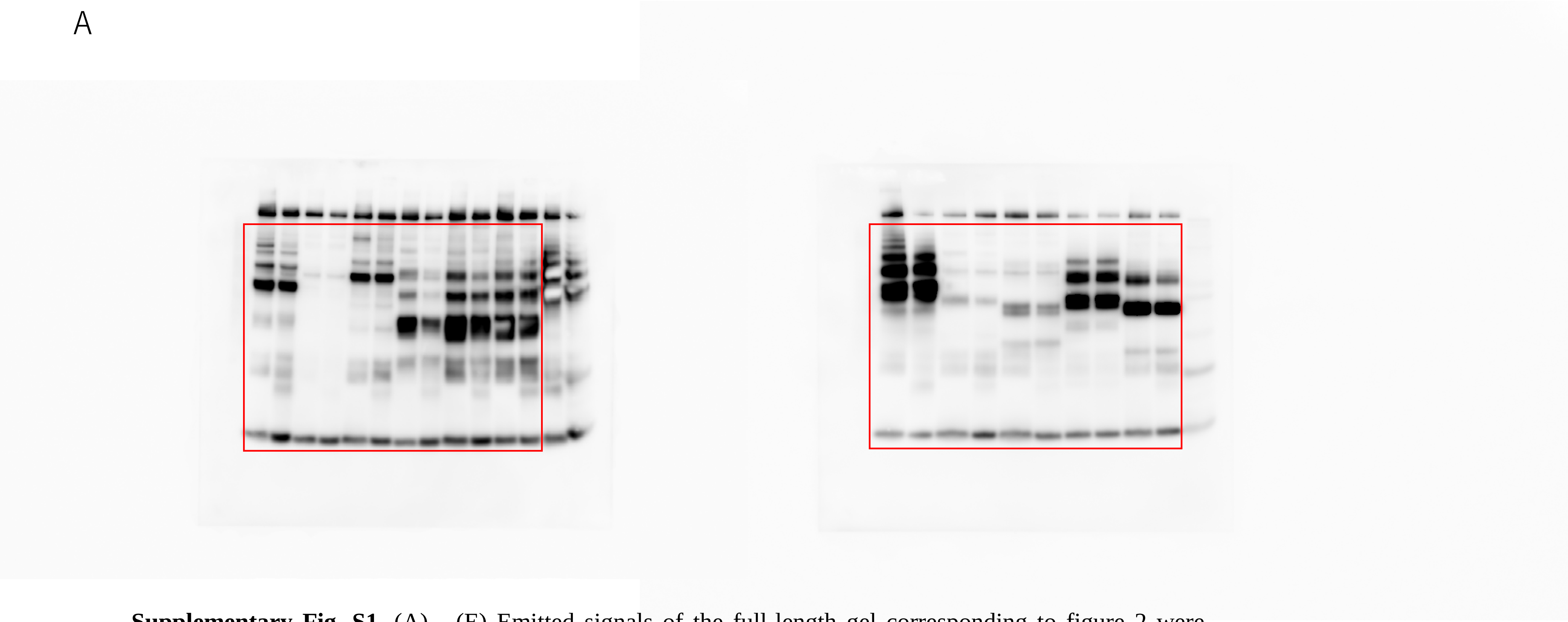

Supplementary Fig. S1. (A) - (E) Emitted signals of the full-length gel corresponding to figure 2 were shown with membrane edges visible. The boxes show the images cropped.

B

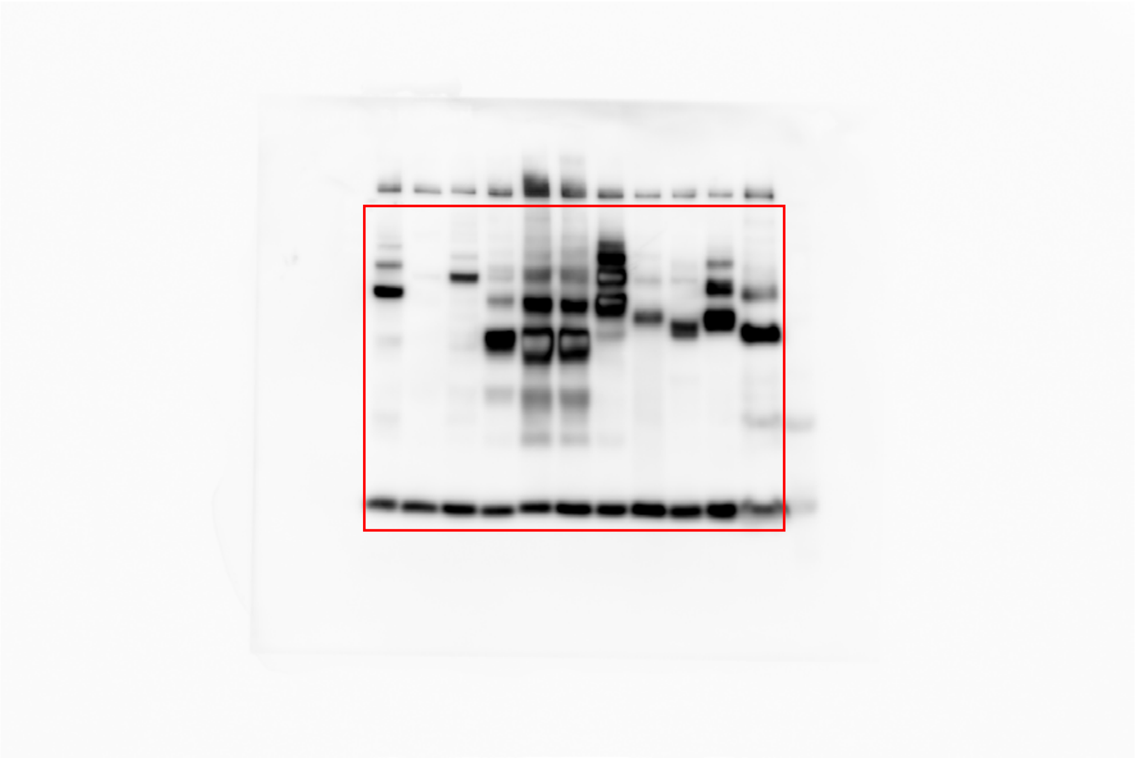

C

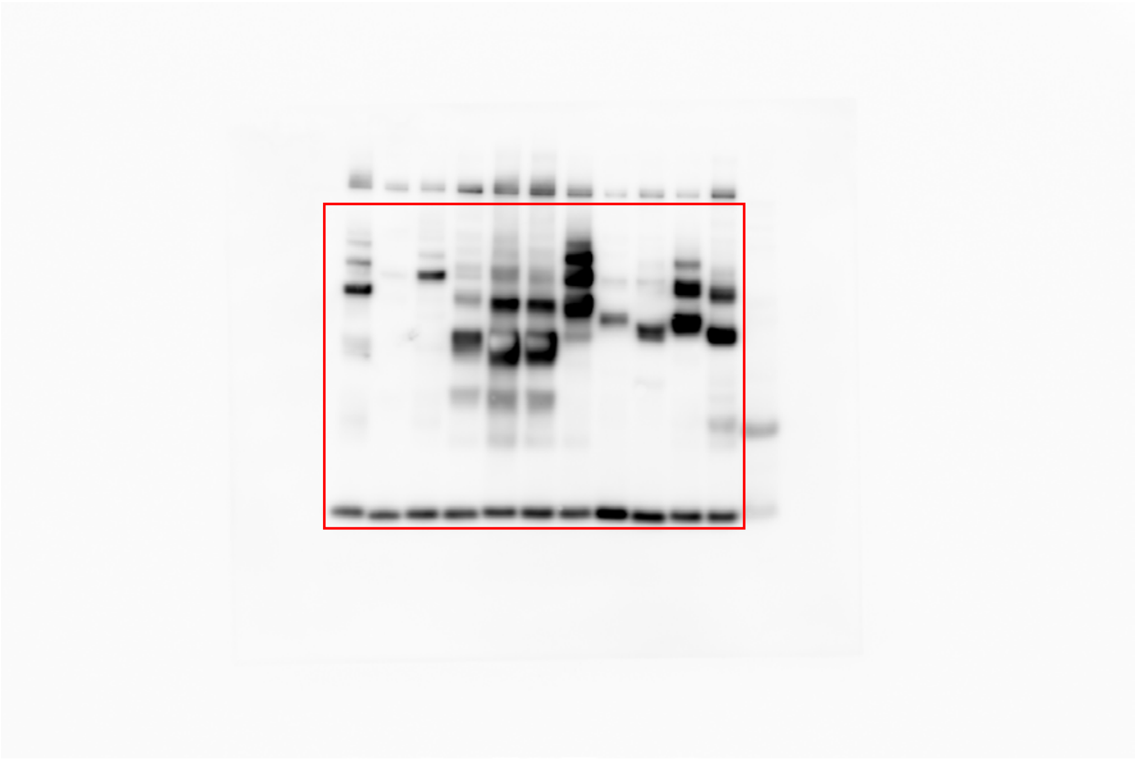

D

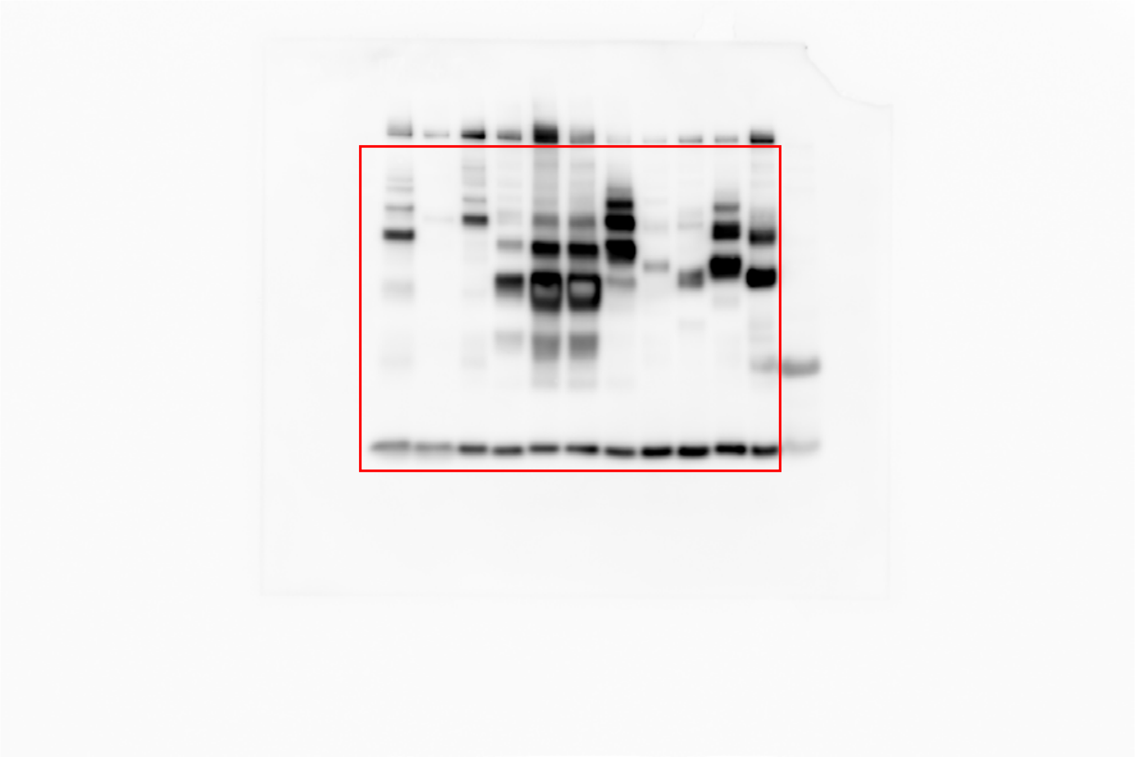

E

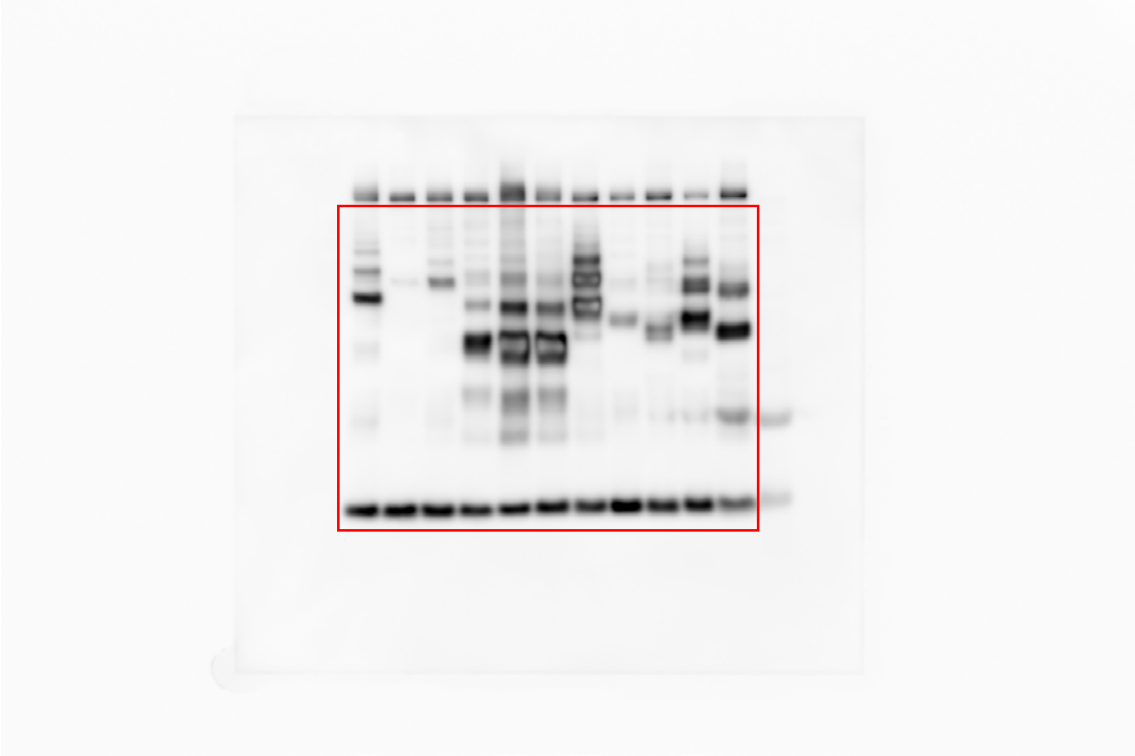

Supplement: Supplementary file 1 — Supplementary Figure S1. [file 41598_2024_52793_MOESM1_ESM.pdf]
