# Supplementary material for: Unique E2-binding specificity of artificial RING fingers in cancer cells
Source: Sci Rep. 2024 Jan 31;14:2545. doi: 10.1038/s41598-024-52793-y (PMC10828389; doi:10.1038/s41598-024-52793-y)
Supplement: Supplementary file 2 — Supplementary Figure S2. [file 41598_2024_52793_MOESM2_ESM.pdf]

Supplementary Fig. S2

A

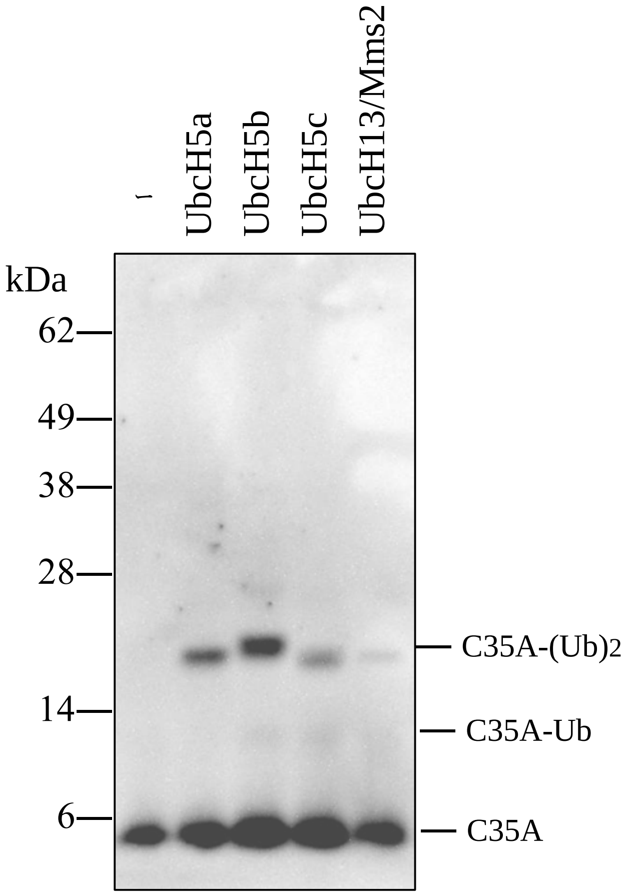

Supplementary Fig. S2. (A) For confirmation of the ubiquitinated products of C35A (replacement of C35 with Ala), randomly biotin-tagged C35A was detected using an avidin-biotin based peroxidase system. The band for the ubiquitinated products cooperated with UbcH5b was distinctly detected, but those for UbcH5a, UbcH5c, and UbcH13 were weak.

B

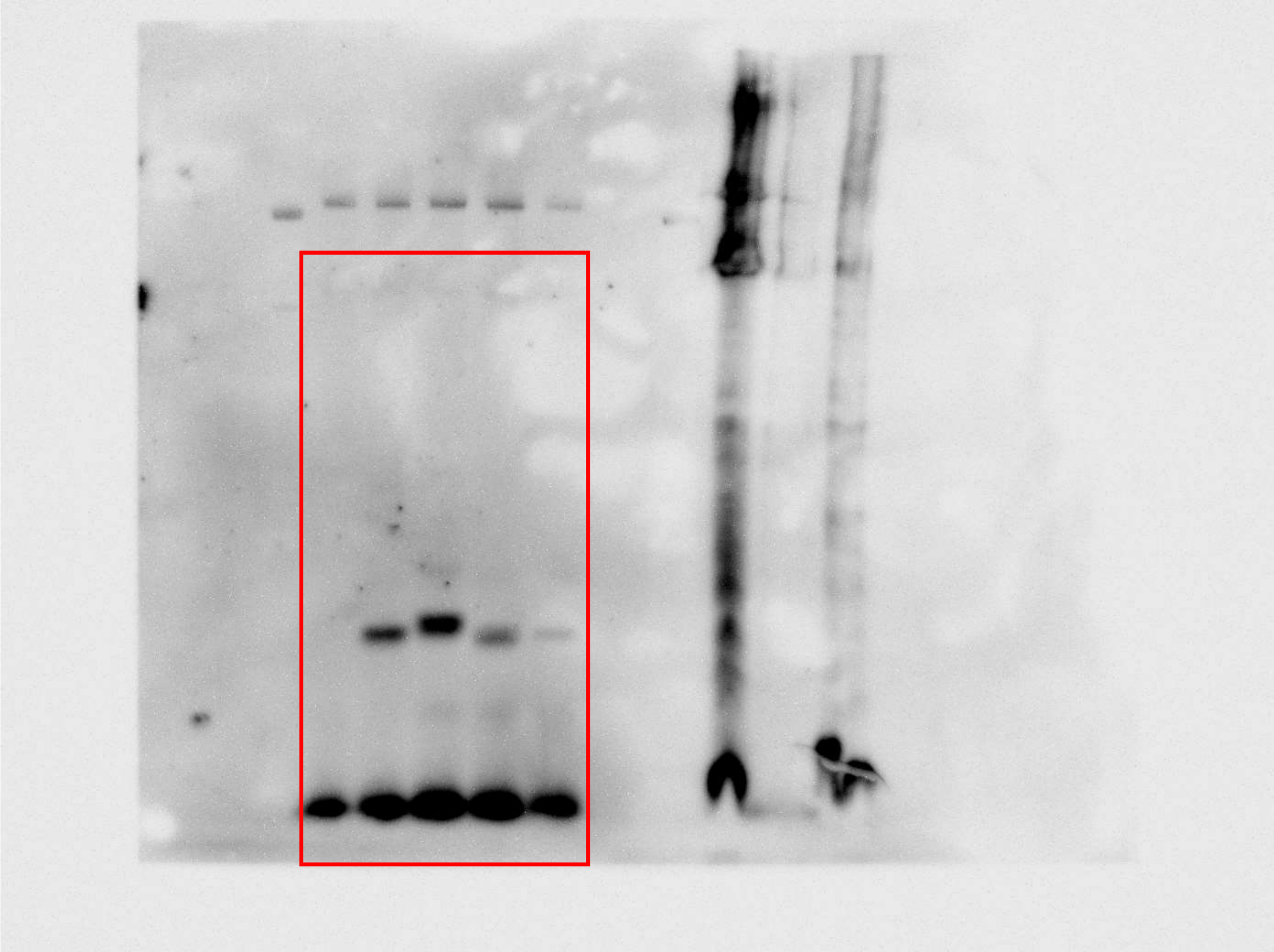

Supplementary Fig. S2. (B) The box shows the image cropped in (A).
