# Supplementary material for: Unique E2-binding specificity of artificial RING fingers in cancer cells
Source: Sci Rep. 2024 Jan 31;14:2545. doi: 10.1038/s41598-024-52793-y (PMC10828389; doi:10.1038/s41598-024-52793-y)
Supplement: Supplementary file 3 — Supplementary Figure S3. [file 41598_2024_52793_MOESM3_ESM.pdf]

Supplementary Fig. S3

A

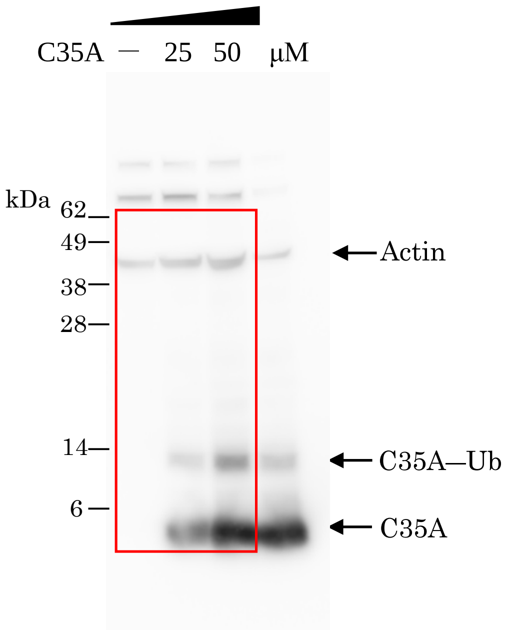

Supplementary Fig. S3. (A) Reactivity of artificial RING finger (ARF) in cancer cells. Emitted signals of the full-length gel corresponding to figure 3 were shown with horseradish peroxidase streptavidin of Vectastain ABC Elite Kits and anti-actin (sc-1616). The box shows the image cropped in Figure 3.

B

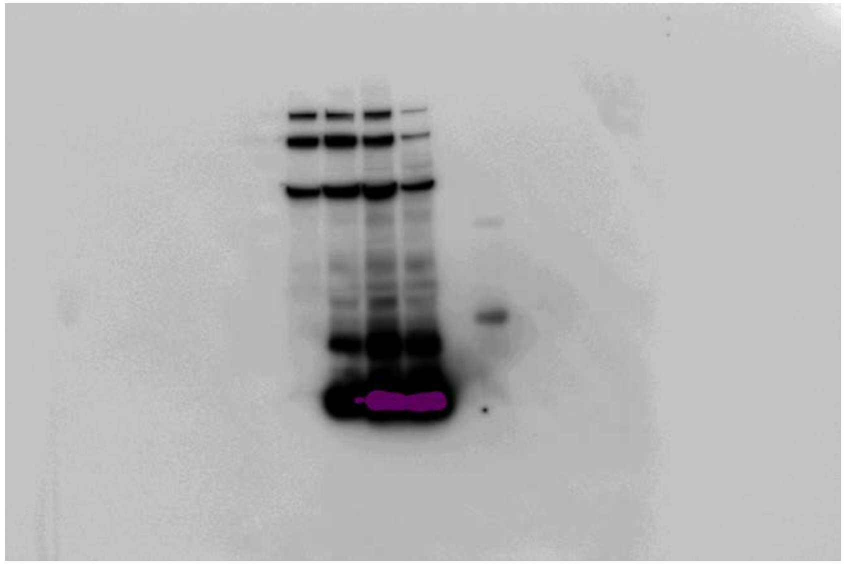

Supplementary Fig. S3. (B) Using a longer exposure time, the emitted signals were detected as in (A). The figure shows the visible edges of the membrane.
