# Supplementary material for: Unique E2-binding specificity of artificial RING fingers in cancer cells
Source: Sci Rep. 2024 Jan 31;14:2545. doi: 10.1038/s41598-024-52793-y (PMC10828389; doi:10.1038/s41598-024-52793-y)
Supplement: Supplementary file 4 — Supplementary Figure S4. [file 41598_2024_52793_MOESM4_ESM.pdf]

Supplementary Fig. S4

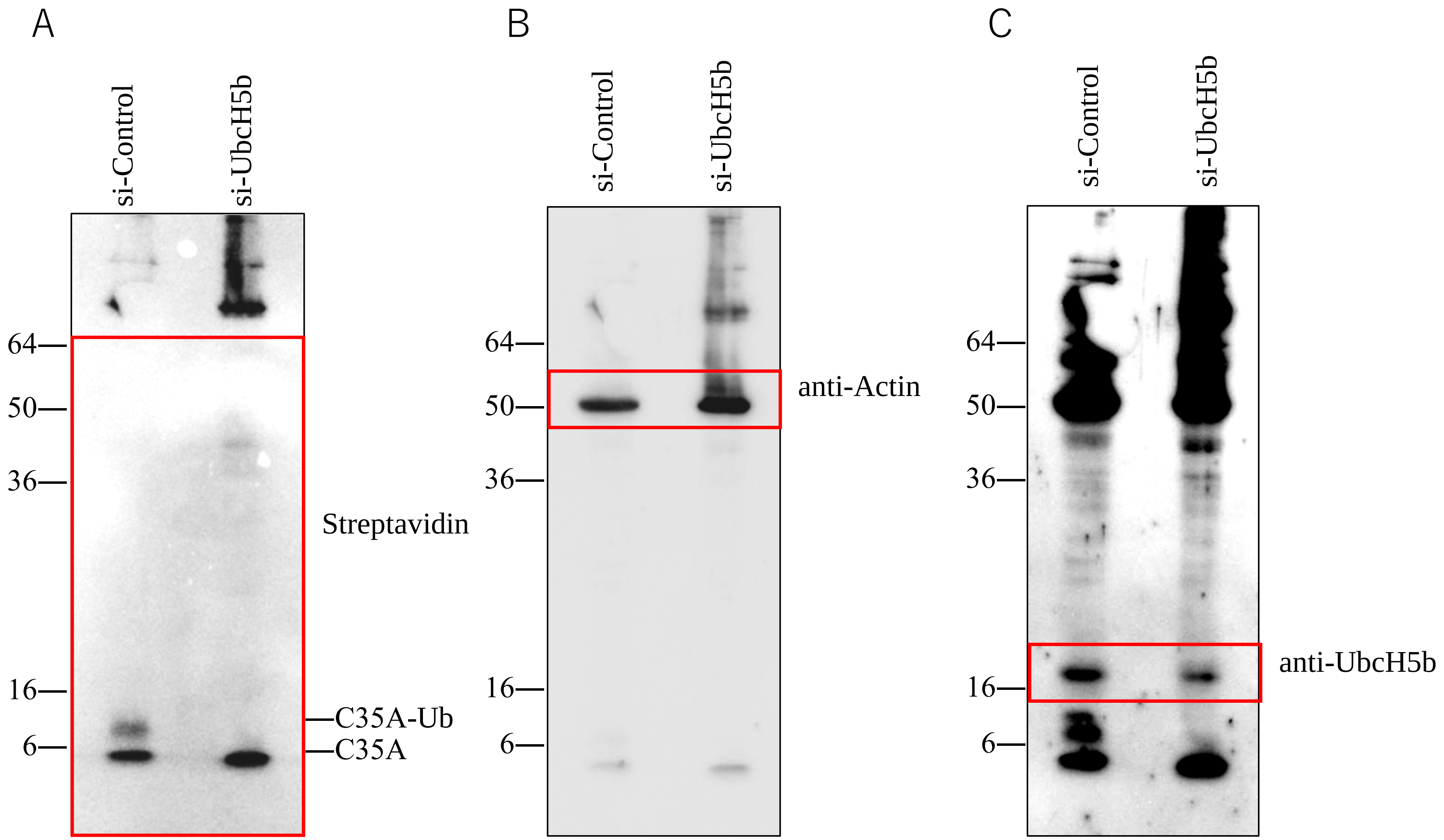

Supplementary Fig. S4. Biotinylated C35A was incubated in UbcH5b-knockdown cells. Emitted signals corresponding to figure 4 were detected in order of A, B, and C with streptavidin (SA00001-0), goat anti-actin (SC-1616), and rabbit anti-UbcH5b (bs-8348), respectively. The boxes show the images cropped in figure 4.

D

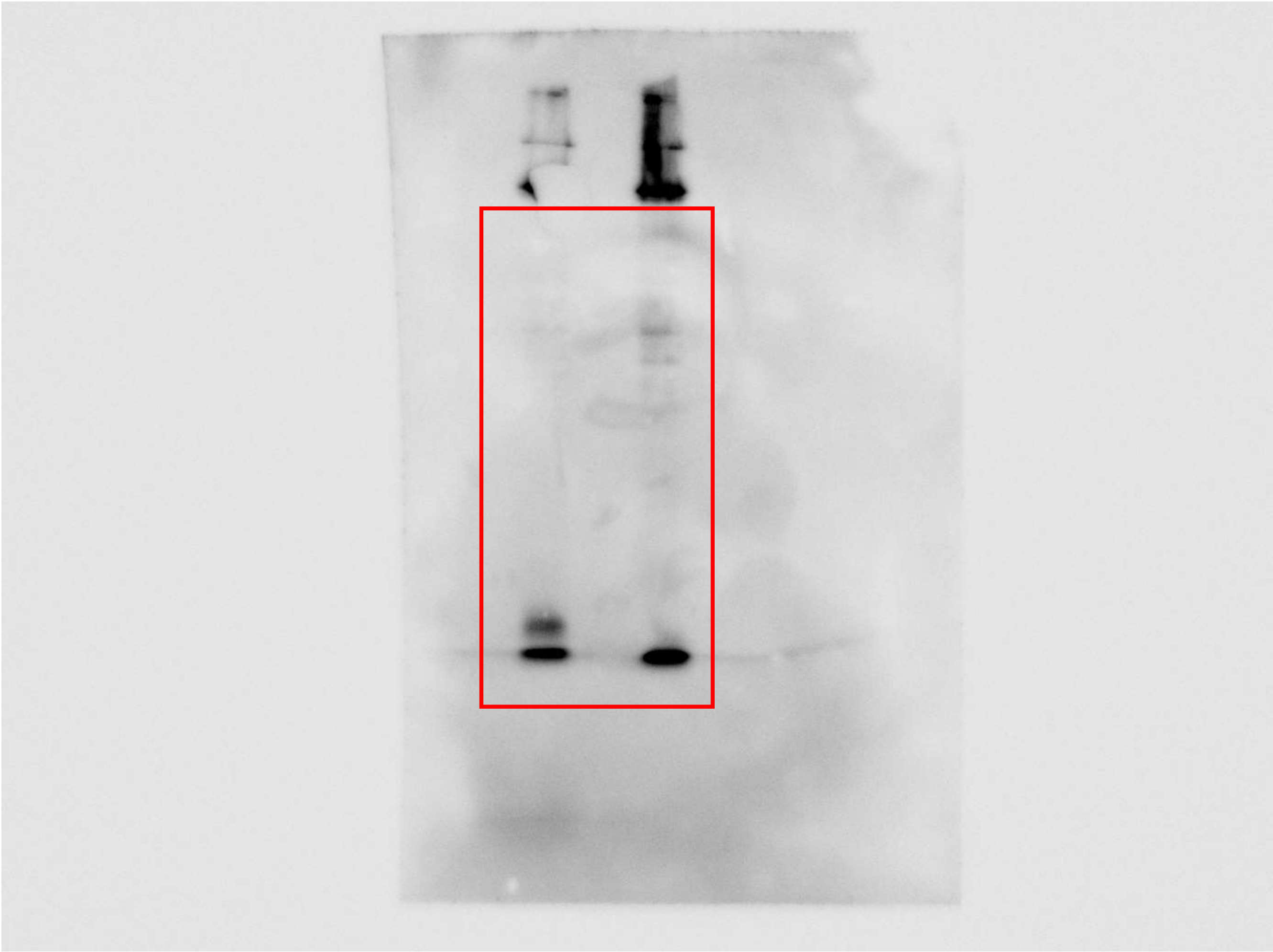

Supplementary Fig. S4. (D) The full-length gel corresponding to the detection with streptavidin in Figure 4 (top panel) was shown with membrane edges visible. The box shows the image cropped.

E

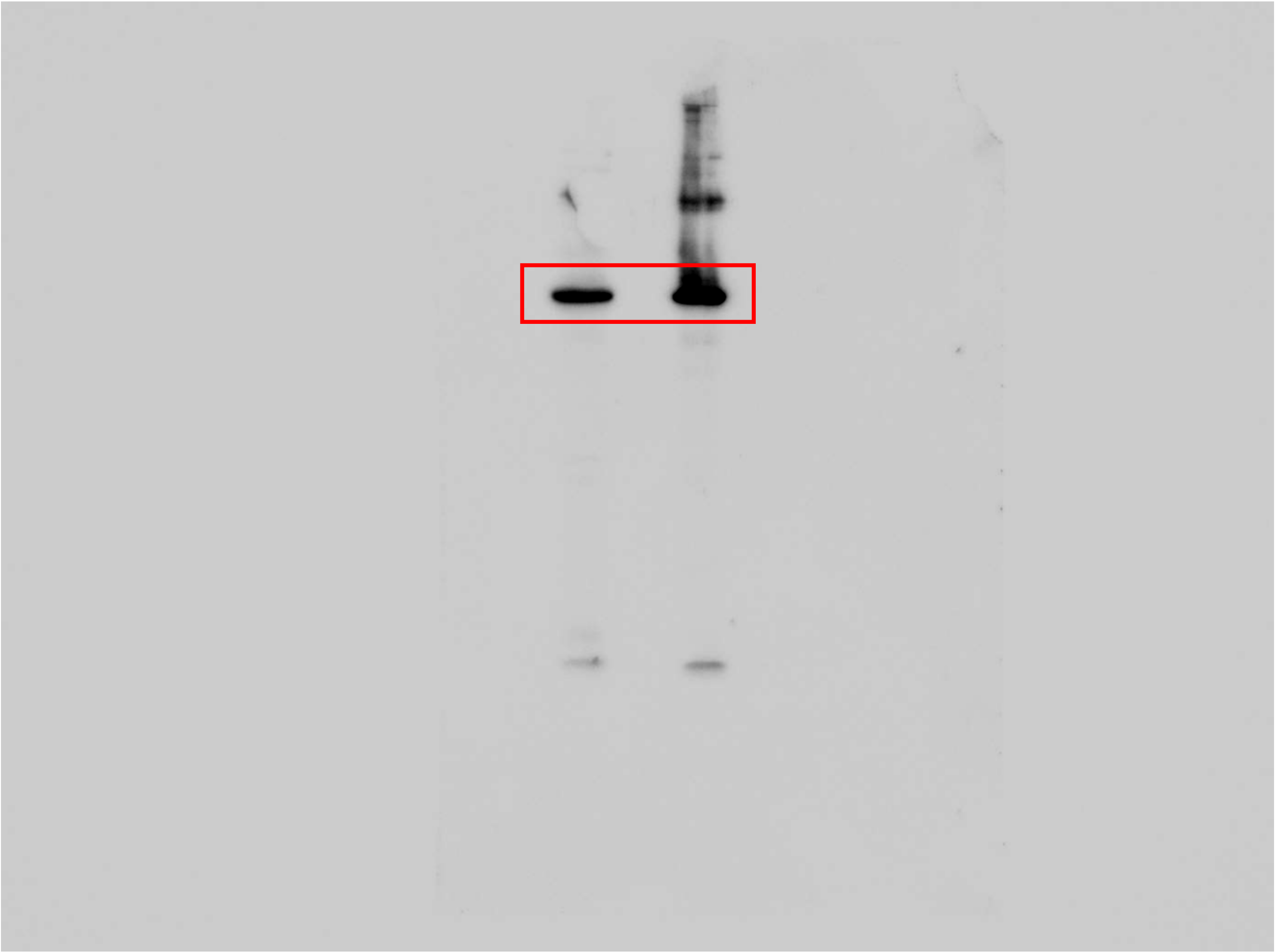

Supplementary Fig. S4. (E) The box shows the image cropped from the full-length gel corresponding to the detection with anti-actin in Figure 4 (bottom panel).

F

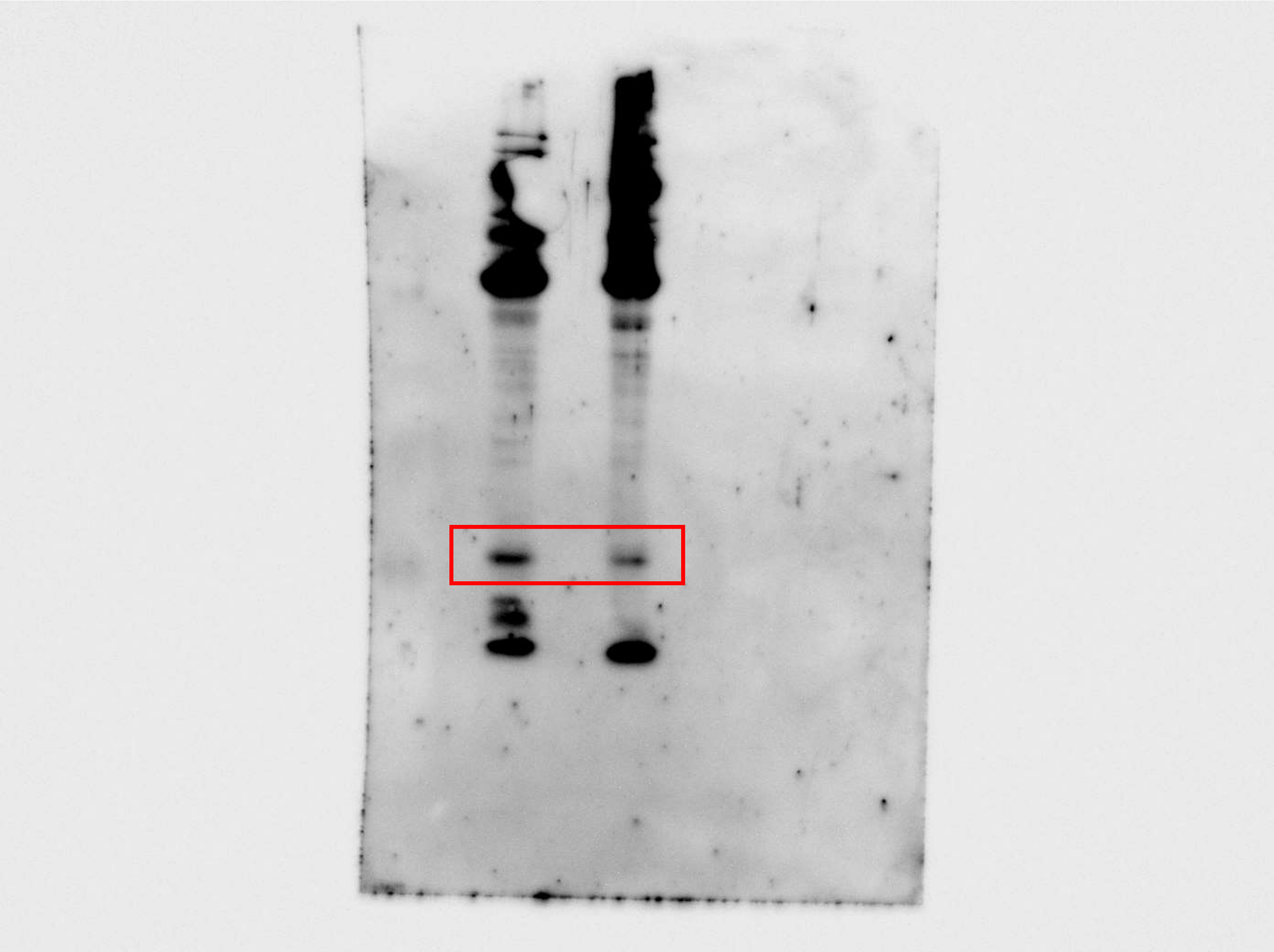

Supplementary Fig. S4. (F) The box shows the image cropped from the full-length gel corresponding to the detection with anti-UbcH5b in Figure 4 (middle panel).
