# Supplementary material for: Unique E2-binding specificity of artificial RING fingers in cancer cells
Source: Sci Rep. 2024 Jan 31;14:2545. doi: 10.1038/s41598-024-52793-y (PMC10828389; doi:10.1038/s41598-024-52793-y)
Supplement: Supplementary file 5 — Supplementary Figure S5. [file 41598_2024_52793_MOESM5_ESM.pdf]

Supplementary Figure

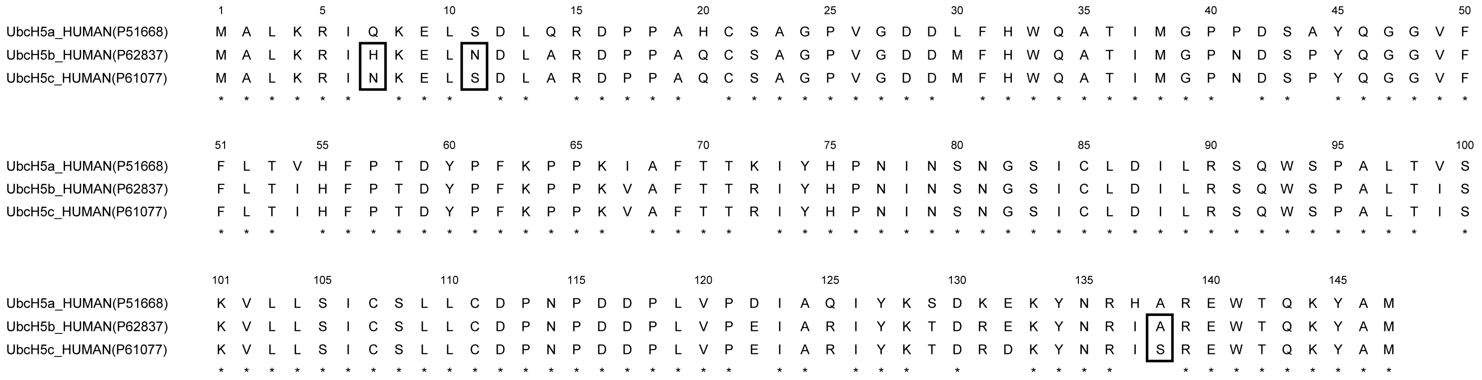

Supplementary Fig. S5. Sequence alignment of E2s (UbcH5a, UbcH5b, and UbcH5c). The different residues between UbcH5b and UbcH5c are residue numbers 7, 11, and 138 which are boxed in the sequence. The stars represent the completely conserved residues.
