# Supplementary material for: Unique E2-binding specificity of artificial RING fingers in cancer cells
Source: Sci Rep. 2024 Jan 31;14:2545. doi: 10.1038/s41598-024-52793-y (PMC10828389; doi:10.1038/s41598-024-52793-y)
Supplement: Supplementary file 6 — Supplementary Figure S6. [file 41598_2024_52793_MOESM6_ESM.pdf]

Supplementary Figure

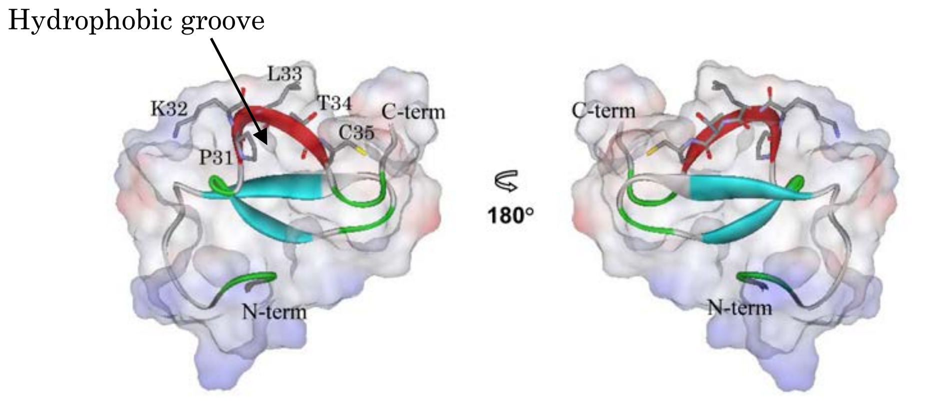

Supplementary Fig. S6. Structure modelling of ARF was built by the program I-TASSER. The ribbon diagrams of the ARF structure were drawn with the side chains of the PKLTC sequence (red). Sheet and turn regions were shown in blue and green, respectively. The Connolly surface was produced as solvent contact areas traced out by a water molecule. The illustrations of the structures were generated using the program Discovery Studio 2.1 (Accelrys Software Inc., San Diego).
